# Relationship between pectoralis major stiffness and shoulder extension range of motion

**DOI:** 10.3389/fphys.2024.1349426

**Published:** 2024-03-06

**Authors:** Andreas Konrad, Marina M. Reiner, Konstantin Warneke, Michael Keiner, Masatoshi Nakamura, Markus Tilp

**Affiliations:** ^1^ Institute of Human Movement Science, Sport and Health, Graz University, Graz, Austria; ^2^ Institute of Sport Science, University of Klagenfurt, Klagenfurt, Austria; ^3^ Department of Exercise Science, German University of Health and Sport, Ismaning, Germany; ^4^ Faculty of Rehabilitation Sciences, Nishi Kyushu University, Saga, Japan

**Keywords:** muscle stiffness, correlation, flexibility, shoulder extension, stiffness

## Abstract

This study aimed to investigate the correlation between the passive muscle stiffness of the pectoralis major muscle pars clavicularis (PMc) and shoulder extension range of motion (ROM) in both male and female participants. Thirty-nine (23 male/16 female) physically active and healthy participants volunteered in this study. After a standardized warm-up, the PMc stiffness was tested via shear wave elastography at a slightly stretched position (long muscle length) and in a non-stretched position (short muscle length). Additionally, a custom-made device and 3D motion capture assessed the active shoulder extension ROM. We found a significant moderate and negative relationship between shoulder extension ROM and PMc stiffness at long muscle length (rs = −0.33; *p* = 0.04) but not at short muscle length (*r* = −0.23; *p* = 0.17). Additionally, there was no significant difference between male and female participants in the correlation analyses at both elbow angles. The moderate correlation between PMc stiffness at a slightly stretched position and shoulder extension ROM suggests that additionally, other structures such as nerves/fascia stiffness or even stretch tolerance might be factors that can be related to shoulder extension ROM.

## 1 Introduction

Muscle stiffness is essential for stabilizing joints and preventing injuries during physical activity, primarily stemming from the inherent mechanical properties of muscle fibers, such as elasticity and viscosity. When a muscle undergoes stretching, its passive tension rises, which restricts the range of motion (ROM) of a joint and acts as a protective mechanism against overextension (Magnusson, 1998). This interplay between muscle stiffness and ROM holds significant importance in both athletic performance and everyday movements. An optimal level of stiffness facilitates effective force generation and transmission, thereby enhancing agility and coordination (Pruyn et al., 2014). Nevertheless, excessive stiffness can impede ROM and disrupt natural movement patterns, elevating the risk of injuries (Butler et al., 2003). Consequently, grasping and regulating muscle stiffness are pivotal for musculoskeletal wellbeing and elevating the overall physical performance.

Several previous studies investigated the relationship between muscle stiffness of lower limb muscles and the ROM of adjacent joints (Miyamoto et al., 2018b; 2018a; Hirata et al., 2020; Nakamura et al., 2021a). Although some studies reported a relationship between lower body muscle stiffness and ROM (Miyamoto et al., 2018a; Hirata et al., 2020), others were not able to confirm these results (Hirata et al., 2020; Nakamura et al., 2021a). More specifically, a relationship between the muscle stiffness of the lower body gastrocnemius medialis (GM) and gastrocnemius lateralis (GL) (including the plantar flexors and hamstring muscles) and ROM (dorsiflexion and hip flexion) has been reported for young participants (Hirata et al., 2020) but not in older participants (Magnusson et al., 1997; Miyamoto et al., 2018a; Hirata et al., 2020; Nakamura et al., 2021a). Apart from an age effect, an effect of sex can be assumed. Miyamoto et al. (2018b) reported a significant relationship between GM + GL stiffness and ankle dorsiflexion ROM in young male participants but not in young female participants. Therefore, many factors seem to influence the ROM–stiffness relationship, including sex, age, and muscle group.

To the best of the authors’ knowledge, there has been no investigation to date, examining the potential correlation between muscle stiffness and ROM in the upper limbs. This inquiry is particularly relevant because shoulder joints, recognized as the most flexible joints in the human body (Halder et al., 2000), are susceptible to injuries. The surrounding muscles and connective tissues play a crucial role in ensuring proper joint positioning, function, and overall performance (Kim et al., 2018). Among the significant muscle groups impacting the shoulder joint as well as the shoulder girdle are the pectoralis muscles. In activities like overhead sports, for instance, tennis, individuals may experience a glenohumeral internal rotation deficit, increasing the risk of shoulder injuries (Le Gal et al., 2018). Possible explanations for this deficit could include diminished muscle length, increased stiffness of the pectoralis muscles, or other soft tissue alterations (Hodgins et al., 2017; Le Gal et al., 2018; Laudner and Thorson, 2019).

Therefore, the study aimed to analyze the relationship between the passive muscle stiffness of the pectoralis major muscle pars clavicularis (PMc) and shoulder extension ROM in both male and female participants. According to the literature on the lower limbs, we hypothesized that the local muscle stiffness is negatively related to the shoulder extension ROM with a moderate to large magnitude. Additionally, we expected sex-specific differences in the correlation analysis.

## 2 Methods

### 2.1 Experimental design

**FIGURE 1:**
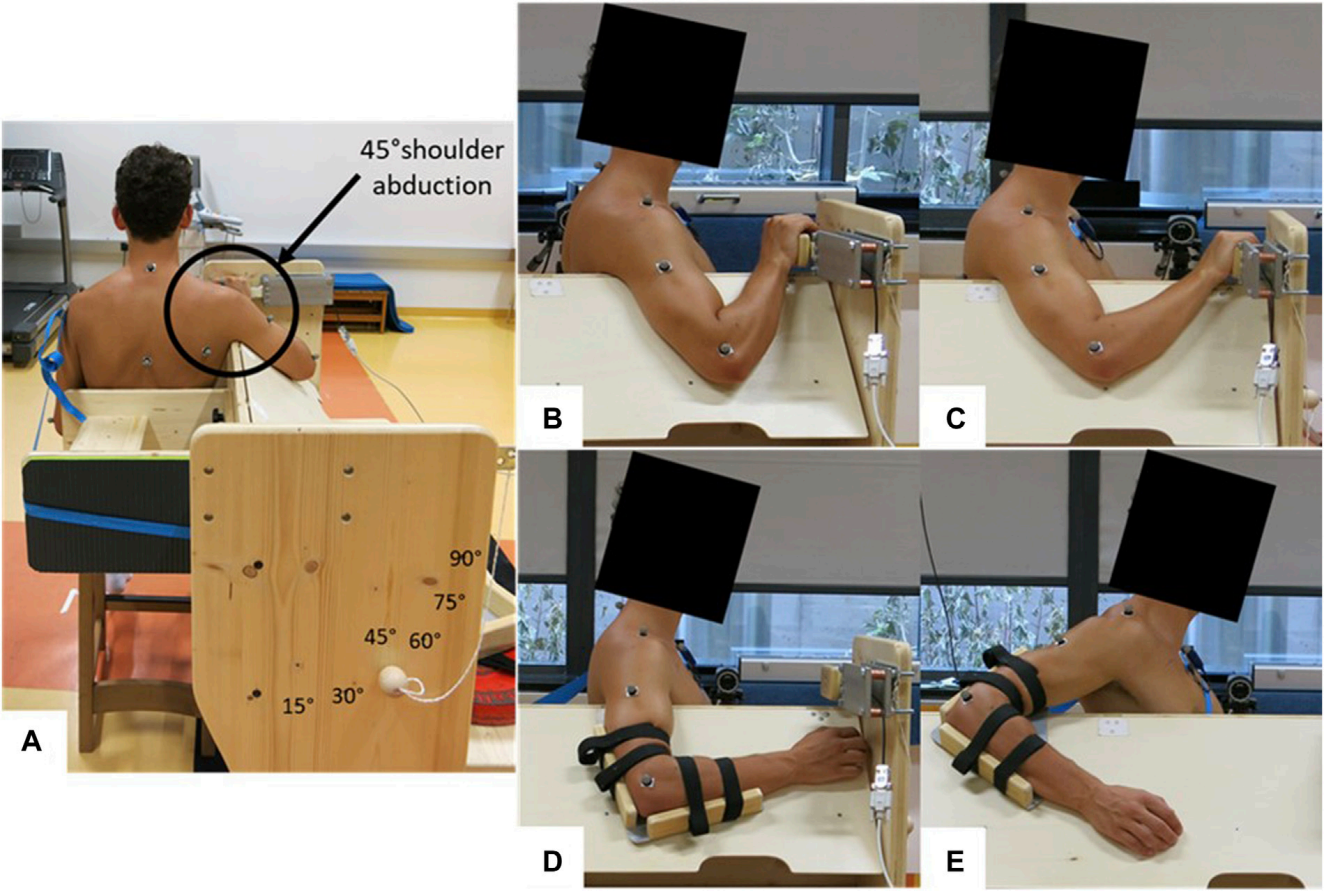
Measurement setup for different tests. **(A)** Back view of the sitting position during all tests; **(B)** measurement position for shear wave elastography at 45° ± 5° elbow flexion and 8° ± 8.6° shoulder flexion (i.e., long muscle length); **(C)** measurement position for shear wave elastography at 90° ± 5° elbow flexion and 31° ± 7.5° shoulder flexion (i.e., short muscle length); **(D)** starting position for the ROM test; **(E)** possible end position during the ROM test.

Each participant attended the laboratory twice, undergoing a familiarization session and a measurement session. Participants were instructed to arrive in a rested state for both sessions, which meant abstaining from high-intensity training for at least 24 h prior to testing. A standardized warm-up consisting of 4 min of parallel arm rotations with both arms (changing direction every minute) at a speed of 120°/s (=20 rotations per minute) was performed by each participant. A metronome provided an auditory signal to maintain the frequency. The measurement side was the PMc and glenohumeral joint of the dominant arm used for writing. The muscle shear modulus of the PMc and shoulder extension ROM were assessed in a seated position at 45° shoulder abduction on a custom-made testing device (Figure 1A). The muscle shear modulus was determined at the elbow flexion angles of 45° ± 5° (i.e., long muscle length) and 90° ± 5° (i.e., short muscle length), resulting in the shoulder flexion angles of 8° ± 8.6° and 31° ± 7.5° [mean ± standard deviation (SD)], respectively (Figures 1B, C). The shoulder extension ROM was conducted with the elbow flexion fixed at 90° (Figures 1D, E). The participant’s position in the custom-made testing device was individually adapted. Care was taken that the participants sat upright and leaned against a backrest, with the investigators ensuring the upright posture of the head, neck, and trunk. Surface electromyography (sEMG) was recorded on the medial part of the PMc to assess muscle activity during all tests. The study received approval from the Ethics Committee of the University of Graz (approval code GZ. 39/4/63 ex 2021/22) and adhered to the standards of the Declaration of Helsinki.

### 2.2 Participants

An *a priori* power analysis, based on the results of a previous study (Hirata et al., 2020), revealed an optimal sample size of 29 participants (correlation: bivariate normal model, pH1 = 0.495, α = 0.05, and β = 0.80). Therefore, to account for potential dropouts, 39 individuals who were healthy and physically active participated in this study. On average, they engaged in weekly training for 16.2 ± 7.5 h. The male participants (*n* = 23) had an average age of 25.6 ± 3.4 years, a height of 183.3 ± 6.8 cm, and a body mass of 81.4 ± 7.1 kg. The female participants (*n* = 16) had an average age of 28 ± 4.5 years, a height of 168.4 ± 5.1 cm, and a body mass of 62.4 ± 7.4 kg. Their sports backgrounds included CrossFit, soccer, overhead sports, or endurance sports. All participants were in good health with no injuries to the glenohumeral joint. Before the familiarization session, participants were briefed on all procedures, and written informed consent was obtained from each participant before their inclusion in the study.

### 2.3 Procedures

#### 2.3.1 Muscle shear modulus

Shear wave elastography (SWE) was conducted using an ultrasound scanner (Aixplorer V12.3, Supersonic Imaging, Aix-en-Provence, France) paired with a linear transducer array (SuperLinear 15–4, 4–15 MHz, Vermont, Tours, France). The measurements were carried out in the SWE mode with specific settings (musculoskeletal preset, penetration mode, smoothing level 5, persistence off, and scale 0–450 kPa). To mark the measurement position on the skin, located midway between the sternomanubrial joint and the beginning of the axillary fold as described by Oliveira et al. (2020), participants stood upright with relaxed arms. During measurements, participants were seated next to a custom-made testing device with the shoulder joint at 45° abduction. In this position, participants generated two elbow flexion angles of 45° ± 5° and 90° ± 5°, resulting in the shoulder flexion angles of 8° ± 8.6° and 31° ± 7.5° (mean ± SD) and, consequently, long (SWE_Long_) and short muscle lengths (SWE_Short_) of the PMc, respectively. The hand rested at shoulder height in front of the participant (Figures 1B, C). A B-mode picture of the initial attempt provided visual support for orientation and reliability during SWE measurements. PMc stiffness in a relaxed state was measured using a handheld technique (Lacourpaille et al., 2012). The ultrasound probe was aligned in the plane with the fascicles and both PMc aponeuroses, and the region of interest (ROI) was defined as large as possible (see Figure 3 of our previous publication; Reiner et al., 2023a). The sEMG signal was visually monitored throughout the measurement to ensure a relaxed muscle state during acquisition. Three 15-s videos were recorded at the marked skin position before and after, and the mean of five consecutive frames with the lowest standard deviation within the ROI was determined for each video (averaged values, analyzed using MATLAB R2017b, MathWorks, Natick, USA; Morales-Artacho et al., 2017). Subsequently, the shear modulus of the PMc for analysis was calculated as the mean between the two closest values of the three videos (Morales-Artacho et al., 2017).

#### 2.3.2 Active shoulder extension ROM

To assess shoulder extension ROM, a 3D motion capture system (Qualisys, Gothenburg, Sweden) was utilized. The participant’s arms and trunk were marked in accordance with the Qualisys “Cast upper body marker set” and supplemented with markers (1 cm diameter) from the “CGM upper body marker set.” Seated beside the testing device at a 45° shoulder abduction, the participant’s elbow angle was securely set at 90° using a custom-made fixation to prevent changes during movement (Figure 1D). To minimize body movements, a strap secured the participant’s trunk to the backrest. The initial position was a neutral shoulder joint position (=0° extension), and participants were instructed to slowly move their arm along a fixed board, extending it as far behind the body as possible while maintaining a low shoulder position (Figure 1E). This movement was repeated three times with 15-s breaks in between. The sEMG of the PMc was continuously recorded during each trial, and if any muscle activation occurred, the attempt was repeated. To determine shoulder joint angles, the recorded markers were mapped onto a model consisting of a torso and upper arm using Visual3D Professional x64 (C-Motion, Inc., Germantown, Virginia, USA). Joint angles in all three planes of motion were then calculated based on the relative positions of the torso and upper arm. The trial with the maximum shoulder extension ROM was selected for further analysis.

### 2.4 Statistical analysis

The data were analyzed using SPSS 26.0. (IBM, Ehningen, DE, Germany). The significance level for all statistical tests was set at *p* < 0.05. The Shapiro–Wilk test for normality was calculated for total and subgroup data. Subgroups were tested for significant differences using the independent *t*-test (for normally distributed data) or Mann–Whitney *U*-test (for non-normally distributed data). If necessary, variance homogeneity was calculated using Levene’s test. Correlations were calculated using Pearson’s correlation coefficient (r) for normally distributed data and using Spearman’s correlation coefficient (rs) for non-normally distributed. Likewise, correlations were calculated for the subgroups but generally using rank correlation (rs) due to the smaller number of cases. The 95% confidence intervals (95% CIs) were calculated for correlation coefficients. Differences in correlation coefficients (after Fisher’s z-transformation) between subgroups (male vs. female) were tested according to Eid et al. (2015) using

$$z=\frac{z_{1}{\;-z}_{2}}{\sqrt[{2}]{\frac{1}{N_{1}-3}+\frac{1}{N_{2}-3}}}.$$

The effect sizes of differences (d) were calculated from *t*-value for the *t*-test as follows:

$$d=t\sqrt{\frac{n_{1}+n_{2}}{n_{1}*n_{2}}}$$

or the z-value

$$r=\frac{z}{\sqrt{n}}\;\text{and}\;d=\frac{2r}{\sqrt{1-r^{2}}}$$

for the Mann–Whitney U-test and differences in correlation coefficients between sexes.

The effect size of the correlation coefficients was determined according to the suggestions of Hopkins (2002) and was defined as trivial (0–0.1), small (0.1–0.3), moderate (0.3–0.5), large (0.5–0.7), very large (0.7–0.9), and nearly perfect or perfect (0.9–1). The inter- and intra-day intraclass correlation coefficients (ICCs, two-way mixed effects model, absolute agreement definition) were calculated for SWE measurements in both elbow positions (long and short muscle lengths). The inter-day ICC was calculated for SWE and ROM between the familiarization session and the data assessment day from a sample of mixed sex obtained from a previous study (Reiner M. M. et al., 2023b).

## 3 Results

The descriptive data of the analyzed variables are presented in Table 1. The SWE intra-day ICC values for the PMc at SWE_Long_ and SWE_Short_ were 0.98 (0.91–0.99) and 0.99 (0.98–0.99), respectively. The SWE inter-day ICC values for the PMc at SWE_Long_, SWE_Short_, and ROM were 0.96 (0.78–0.99), 0.89 (0.42–0.98), and 0.95 (0.80–0.99), respectively. The calculation results for the total group using the Shapiro–Wilk test showed that ROM and SWE_Short_ were normally distributed and that SWE_Long_ data were not normally distributed. Similarly, for the male and female subgroups, the distribution of variables was analogous to the total group. Levene’s test showed homogeneous variances for normally distributed variables (total group; *p* > 0.05). ROM (t = 1.26; *p* = 0.215; d = 0.41), SWE_Long_ (z = 0.329; *p* = 0.748; d = 0.14), and SWE_Short_ (t = 0.02; *p* = 0.39; d = 0.01) showed no significant differences between male and female participants.

**TABLE 1:** Mean and standard deviation of the analyzed variables for the total and subgroups (i.e., male and female participants).

	Male (n = 23)	Female (n = 16)	Total group (n = 39)
**ROM (°)**	67.6 ± 6.5	70.5 ± 7.9	68.8 ± 7.1
**SWE**_**Long**_ **(kPa)**	9.5 ± 2.5	11.0 ± 5.3	10.1 ± 3.9
**SWE**_**Short**_ **(kPa)**	7.5 ± 2.7	7.5 ± 2.2	7.5 ± 2.5

ROM, range of motion; SWE_Long_, shear wave elastography in long muscle length; SWE_Short_, shear wave elastography in short muscle length.

**FIGURE 2:**
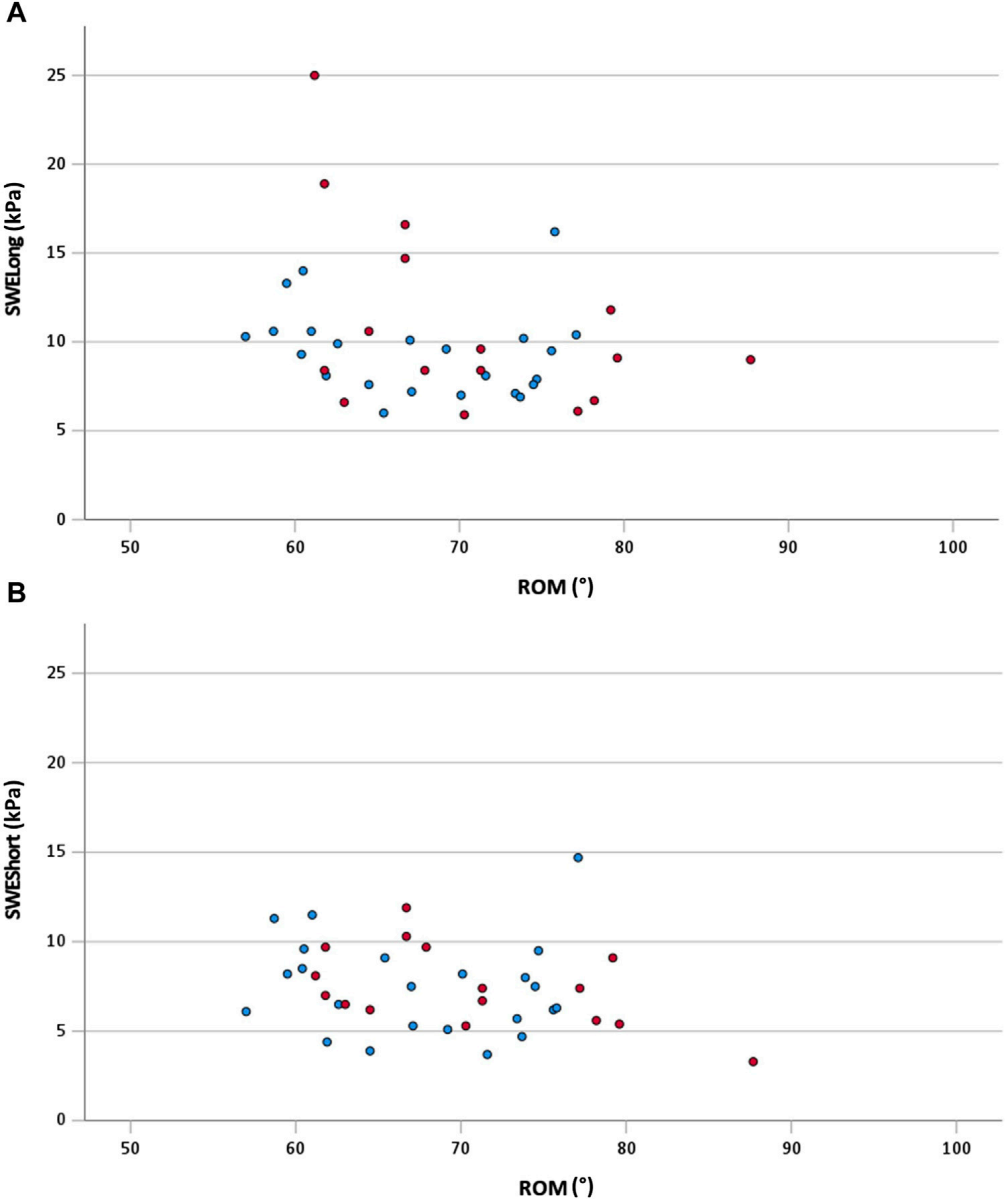
**(A)** Scatterplot of ROM and SWE_Long_ for the total sample with r_s_ = −0.33 (95% CI: −0.62 to −0.04; *p* = 0.04); **(B)** scatterplot of ROM and SWE_Short_ for the total sample with r = −0.23 (95% CI: −0.54 to 0.09; *p* = 0.17) red dots, female participants; blue dots, male participants; ROM, range of motion; SWE_Long_, shear wave elastography at long muscle length; SWE_Short_, shear wave elastography at short muscle length.

The correlations for the total sample are shown in Figure 2. Between ROM and SWE_Long_ (total sample), a significant moderate correlation was shown (rs = −0.33; 95% CI: −0.62 to −0.04; *p* = 0.04). The other correlations for the total group remained non-significant (*p* > 0.05). The correlations of the subgroups are shown in Table 2. For both subgroups, no significant (*p* > 0.05) correlations were calculated between the analyzed variables. Additionally, no significant (*p* > 0.05; d = 0.08–0.3) differences in correlation coefficients were calculated between the subgroups.

**TABLE 2:** Spearman’s correlation coefficient, 95% CIs, and differences in the correlation coefficient for subgroups.

Variable	Male	Female	Differences in the correlation coefficient
**ROM–SWE**_**Long**_	−0.25 (*p* = 0.25)	−0.33 (*p* = 0.21)	n.s
	95% CI (−0.66 to 0.16)	95% CI (−0.82 to 0.15)	z = 0.25; *p* = 0.80; d = 0.08
**ROM–SWE**_**Short**_	−0.11 (*p* = 0.61)	−0.43 (*p* = 0.09)	n.s
	95% CI (−0.54 to 0.32)	95% CI (−0.87 to 0.10)	z = 0.98; *p* = 0.33; d = 0.32

ROM, range of motion; SWE_Long_, shear wave elastography in long muscle length; SWE_Short_, shear wave elastography in short muscle length; n.s, not significantly different between subgroups; 95% CI, 95% confidence interval.

## 4 Discussion

The purpose of this study was to analyze the possible relationship between the passive muscle stiffness of PMc assessed via shear wave elastography and the shoulder extension ROM and to analyze the potential sex differences in these relationships. Although the ICCs of the assessed methods can be classified as high (Vincent and Weir, 2012), we found a significant moderate and negative relationship between the shoulder extension ROM and PMc stiffness at long muscle length (but not at short muscle length) for the whole sample (i.e., male and female participants). For at least the muscle stiffness at long muscle length, this result might indicate that higher stiffness values led to the lower shoulder extension ROM. Additionally, we did not find a significant difference between male and female participants in the correlation analyses at short and long muscle lengths.

Previous similar studies have conducted experiments on the lower limbs only. Miyamoto et al. (2018a) reported a significant low to moderate relationship between the sit-and-reach score and the muscle stiffness of the semimembranosus, biceps femoris, and semitendinosus, as well as overall hamstring stiffness (*r* = −0.25 to −0.33). Additionally, Miyamoto et al. (2018b) and Hirata et al. (2020) found significant large and small relationships between GM and GL stiffness and ankle dorsiflexion ROM at a slightly stretched position in young male participants [(Miyamoto et al., 2018b) 14° plantarflexion (*r* = −0.51 to −0.62); (Hirata et al., 2020) 15° plantarflexion (*r* = −0.495 to −0.664)]. In a neutral and therefore non-stretched position (0° plantarflexion), Miyamoto et al. (2018b) reported no significant relationship between the stiffness of GM and ankle dorsiflexion ROM but revealed a significant relationship between GL stiffness and ankle dorsiflexion ROM (*r* = −0.61). Similarly, our study showed a significant relationship between the shoulder extension ROM and PMc stiffness at long muscle length but not in a position where the PMc was not stretched (i.e., short muscle length).

Considering the effect size of the correlation, we have found a moderate relationship between shoulder extension ROM and PMc stiffness at long muscle length only (rs = −0.33). This is in line with the findings of Miyamoto et al. (2018a) who even reported weak relationships between the sit-and-reach score and the stiffness of different single-hamstring muscles and a moderate relationship between the sit-and-reach score and the overall hamstring stiffness. Remarkable in our findings is two female outliers at SWE_Long_ with 18.9 and 25.0 kPa (Figure 2A). Removing these two female outliers (i.e., sensitivity analysis) changes the relationship (rs = −0.24) between ROM and SWE_Long_ to a negligible and even insignificant relationship (*p* = 0.15). Consequently, it can be assumed that additional factors like the stiffness of other muscles or non-muscular structures, such as fascia, nerves, or capsules (Freitas et al., 2018), and the contribution of antagonist muscles might be responsible for joint restriction in terms of ROM. Additionally, likely neuronal mechanisms such as stretch tolerance (Magnusson et al., 1997) are related to the ROM of a joint.

Muscle stiffness is affected by interventions like stretching, which was consistently shown in the lower limbs (Konrad et al., 2017; Takeuchi et al., 2023). For the upper limbs, a decrease in the muscle stiffness of the pectoralis muscle (i.e., pectoralis minor) assessed with SWE was reported following a single bout of 30 s of stretching of the pectoralis muscles (Umehara et al., 2021). Although these authors did not assess ROM in their study, it is well known that a single bout of stretching can induce changes in ROM (Behm et al., 2016; Konrad et al., 2019). Consequently, it can be assumed that in the study by Umehara et al. (2021), an increase in the shoulder extension ROM appeared after the single stretching exercise, and consequently, the change in ROM can be likely explained in part by the decrease in muscle stiffness. Following a long-term stretching protocol mainly in the lower body muscles, recent meta-analyses reported an increase in ROM (Konrad et al., 2023) and a decrease in muscle stiffness (Takeuchi et al., 2023), indicating a causal relationship. In contrast, when considering the upper body, a 7-week stretching exercise of the pectoralis muscle led to an increase in ROM, however, without a change in PMc stiffness (Reiner M. et al., 2023a). It was assumed by the authors that the stiffness of other structures such as tendons, ligaments, and capsules or even other muscles might have changed throughout the stretching protocol and have consequently contributed to the ROM increase. In summary, the results of at least some studies (Konrad et al., 2017; Nakamura et al., 2021b; Takeuchi et al., 2023) indicate that a decrease in lower leg muscle stiffness leads to a higher ROM.

ROM is sex-dependent with female populations having greater flexibility than male populations (Medeiros et al., 2013; Konrad et al., 2022), which is especially required for the female populations at the time of childbirth. Interestingly, a significant relationship between GM and GL stiffness and ankle dorsiflexion was only shown in male participants but not in female participants (Miyamoto et al., 2018a). Consequently, it was assumed that based on the results of Miyamoto et al. (2018a) for GM and GL, the relationship between the stiffness of other muscle groups and the respective joint ROM might be sex-dependent as well. However, this was not supported by the findings of the present study. In a previous study, we have found a significant difference in joint (i.e., muscle–tendon unit) stiffness of the plantar flexors (Konrad et al., 2017) between male and female participants. However, the higher joint stiffness in male participants compared to female participants could not be explained by the differences in muscle stiffness but rather by significantly higher Achilles tendon stiffness in male participants. Similarly, the muscle stiffness of the PMc in our experiment was similar between male and female participants according to our analyses (Table 1). Regarding ROM, female participants showed non-significant higher values of the shoulder extension ROM (70.5°) compared to male participants (67.6°). In general, it was reported that in most of the joint stiffness, female participants have higher ROM values compared to male participants (Medeiros et al., 2013). However, even if the female participants show higher ROM values compared to male participants, similar muscle stiffness values do not change the slope of the relationships between ROM and muscle stiffness. Accordingly, this might explain why we did not find a significant difference in the relationship between PMc stiffness and shoulder extension ROM between male and female participants.

This study has some limitations. First, our participants had different sports backgrounds such as CrossFit, soccer, overhead sports, or endurance sports. Consequently, there was likely a heterogeneity that might have influenced our results. Due to the smaller sample sizes within these specific sports groups, it was not possible to detect potential between-group differences. Second, although the overall sample was sufficient based on our sample size calculation, there might have been very little power for detecting sex-specific relationships. Additionally, the number of female participants (*n* = 16) was a bit lower compared to that of the male participants (*n* = 23), which might have influenced our findings.

## 5 Conclusion

It can be concluded that there is a moderate relationship between PMc stiffness at long muscle length in a slightly stretched position and shoulder extension ROM. However, we have not found any significant relationship between PMc stiffness at short muscle length and shoulder extension ROM. Additionally, we have not found any significant difference between male and female participants. The moderate correlation between SWE_Long_ and shoulder extension ROM suggests that stiffness of other structures such as nerves/fascia or even stretch tolerance might be factors that can be related to shoulder extension ROM.

## Data Availability

The raw data supporting the conclusion of this article will be made available by the authors, without undue reservation.
